# Pituitary Gland Metastases As the Initial Presentation of Lung Adenocarcinoma: A Case Report

**DOI:** 10.7759/cureus.94841

**Published:** 2025-10-18

**Authors:** Angela E Russo, Hannah Johnson, Ximena Jordan Bruno, Christopher J Anker, Hibba Rehman

**Affiliations:** 1 Internal Medicine, University of Vermont Medical Center, Burlington, USA; 2 Pediatrics, University of Vermont, Burlington, USA; 3 Oncology, Perelman Center for Advanced Medicine, Philadelphia, USA; 4 Radiation Oncology, University of Vermont Medical Center, Burlington, USA; 5 Oncology, University of Vermont Medical Center, Burlington, USA

**Keywords:** chemoradiation therapy, metastatic non-small cell lung cancer, neuroimaging studies, pituitary metastasis, visual field defect

## Abstract

Pituitary gland metastases (PMs) are rare and typically asymptomatic, often discovered incidentally in patients with known malignancies. Symptomatic PMs as the initial presentation of cancer are uncommon. We report a case of a 52-year-old woman with no prior oncologic history who presented with progressive unilateral vision loss, headaches, and systemic symptoms. Imaging revealed a sellar and suprasellar mass, and endocrine evaluation indicated both anterior and posterior pituitary dysfunction. Surgical resection and histopathological analysis confirmed metastatic adenocarcinoma of the lung. The patient was treated with radiation to the sellar region and systemic chemoimmunotherapy, resulting in radiologic improvement and partial improvement in vision four months after the initiation of treatment. This case underscores the importance of considering metastatic disease in the differential diagnosis of pituitary masses, particularly in patients with acute neuro-ophthalmologic and endocrine abnormalities. Though difficult to distinguish clinically and radiologically from primary sellar tumors, the presence of diabetes insipidus may indicate a PM diagnosis given its rarity in pituitary adenoma cases. Early recognition and multidisciplinary intervention are critical to improving outcomes and preserving function in such rare and complex presentations.

## Introduction

The pituitary gland is a rare site of metastasis. When pituitary metastases are identified, they are often asymptomatic, with a historical rate of only 7% reported as symptomatic [1], and are found on incidental imaging of oncology patients. When symptomatic, diabetes insipidus is the single most common symptom, presenting in 32.76%-42.34% of cases [2]. Other symptoms include visual defects (27.89%-41.38%), cranial nerve palsies (21.58%-41.38%), and headaches (15.79%-32.76%) [2].

The origin of metastasis to the pituitary gland (PM) has been reported to be most commonly breast (25%), lung (18.75%), and renal cell carcinoma (14.6%) [3]. In a large registry of nearly 18,000 pituitary tumor cases, only 0.5% are PMs [3]. However, the true incidence is likely higher than that, with 1.9% PMs reported among cancer patient autopsy cases [2]. Nevertheless, there are a few reported cases describing symptomatic PMs in patients with no known malignancy.

We present here an uncommon case of a patient showing signs and symptoms of pituitary gland involvement as the initial presentation of her cancer.

## Case presentation

A 52-year-old woman with a past medical history pertinent to alcohol abuse, heavy smoking (over 40 pack-years), depression, anxiety, and hypertension presented with one month of moderate headaches, mild nausea, 10 days of rapidly progressive unilateral right eye visual loss, and one week of chills and night sweats. Her right eye had a relative afferent pupillary defect, loss of light perception and color vision, and a loss of the majority of visual fields except for intact vision to hand motion superonasally and a single focus of intact vision approximately five degrees in diameter. There was no acute process noted, such as atrophy. Vision in the left eye was normal, and other cranial nerves were intact. Labs revealed hyperprolactinemia, low adrenocorticotropic hormone, central hypothyroidism, and diabetes insipidus (Table 1).

**Table 1:** Patient lab values with reference ranges ACTH: adrenocorticotropic hormone; TSH: thyroid-stimulating hormone

Lab	Patient result	Reference range
Prolactin	69.2 (high)	Nonpregnant: 2.8-29.2; pregnant: 9.7-208.5; postmenopausal: 1.8-20.3
ACTH	<5.0 (low)	7.2-63 (a.m. collection)
TSH	2.42 (normal)	0.47-4.68 uIU/mL
T3	87 (low)	97-169 ng/dL
T4	3.7 (low)	5.5-11.0 ug/dL
Sodium	130 (low)	136-145 mEq/L

**Figure 1:**
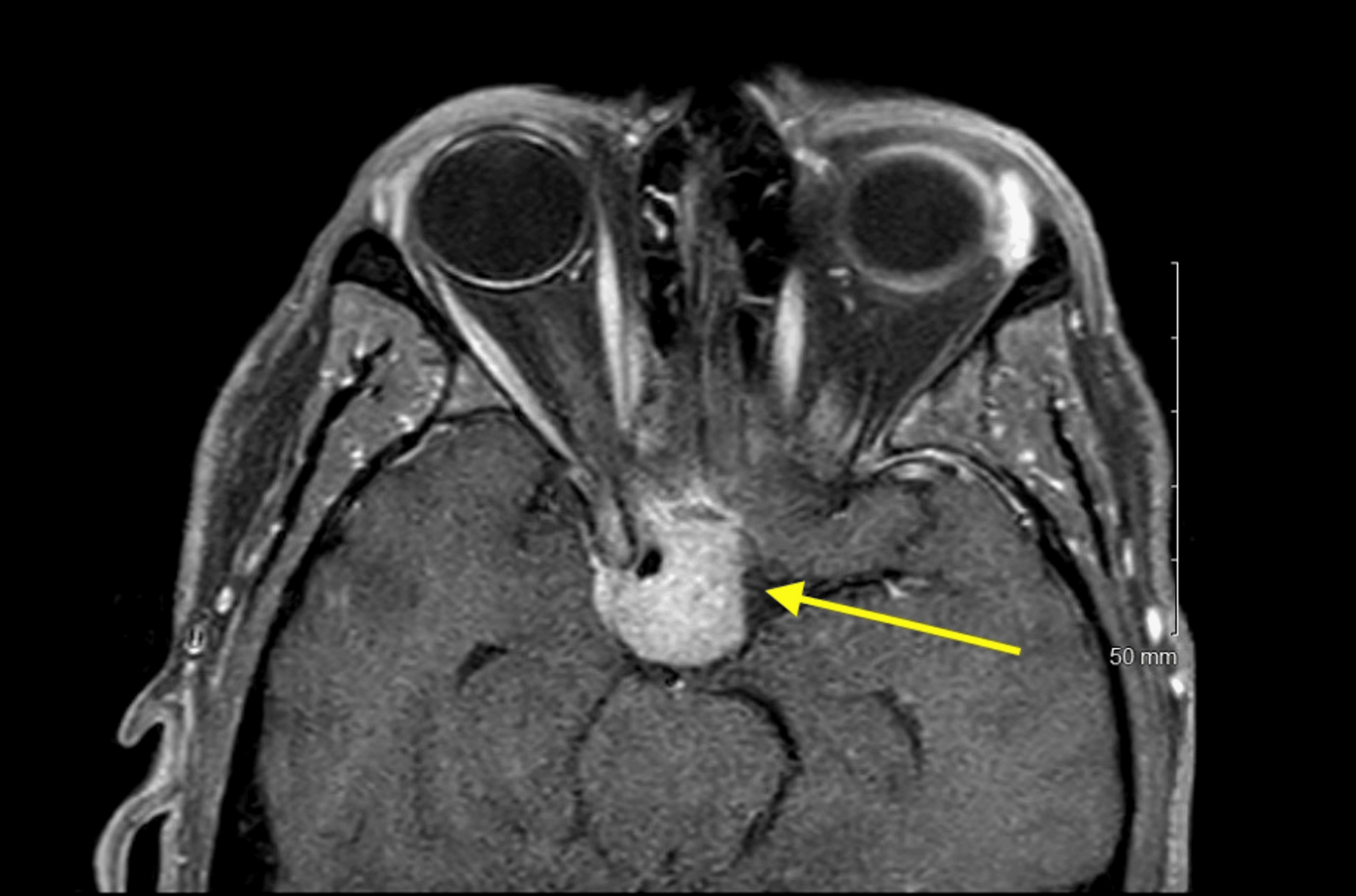
Axial T1 MRI of the orbits with intravenous gadolinium contrast and fat suppression series showing slight extension of tumor into the right cavernous sinus and tumor contacting the prechiasmatic optic nerves

**Figure 2:**
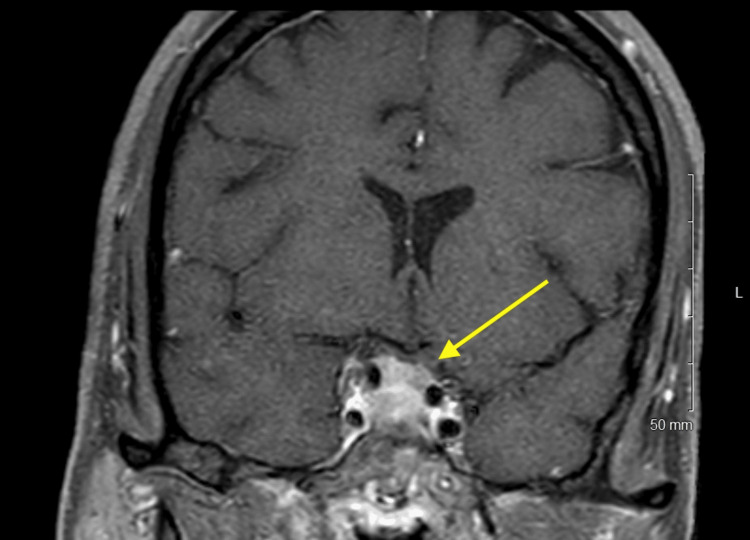
Coronal T1 MRI of the orbits with intravenous gadolinium contrast and fat suppression series showing slight extension of tumor into the right cavernous sinus and tumor contacting the prechiasmatic optic nerves

MRI revealed an expansile sellar and suprasellar mass measuring approximately 22 mm × 20 mm × 19 mm; notable features included slight extension of tumor into the right cavernous sinus, tumor surrounding the paraclinoid segment of the right internal carotid artery, tumor contacting the inferomedial aspect of the prechiasmatic optic nerves, and extension along the superior dorsal surface of the clivus and contacting the distal ventral surface of the basilar artery (Figures 1, 2). MRI of the head did not show any other metastases.

**Figure 3:**
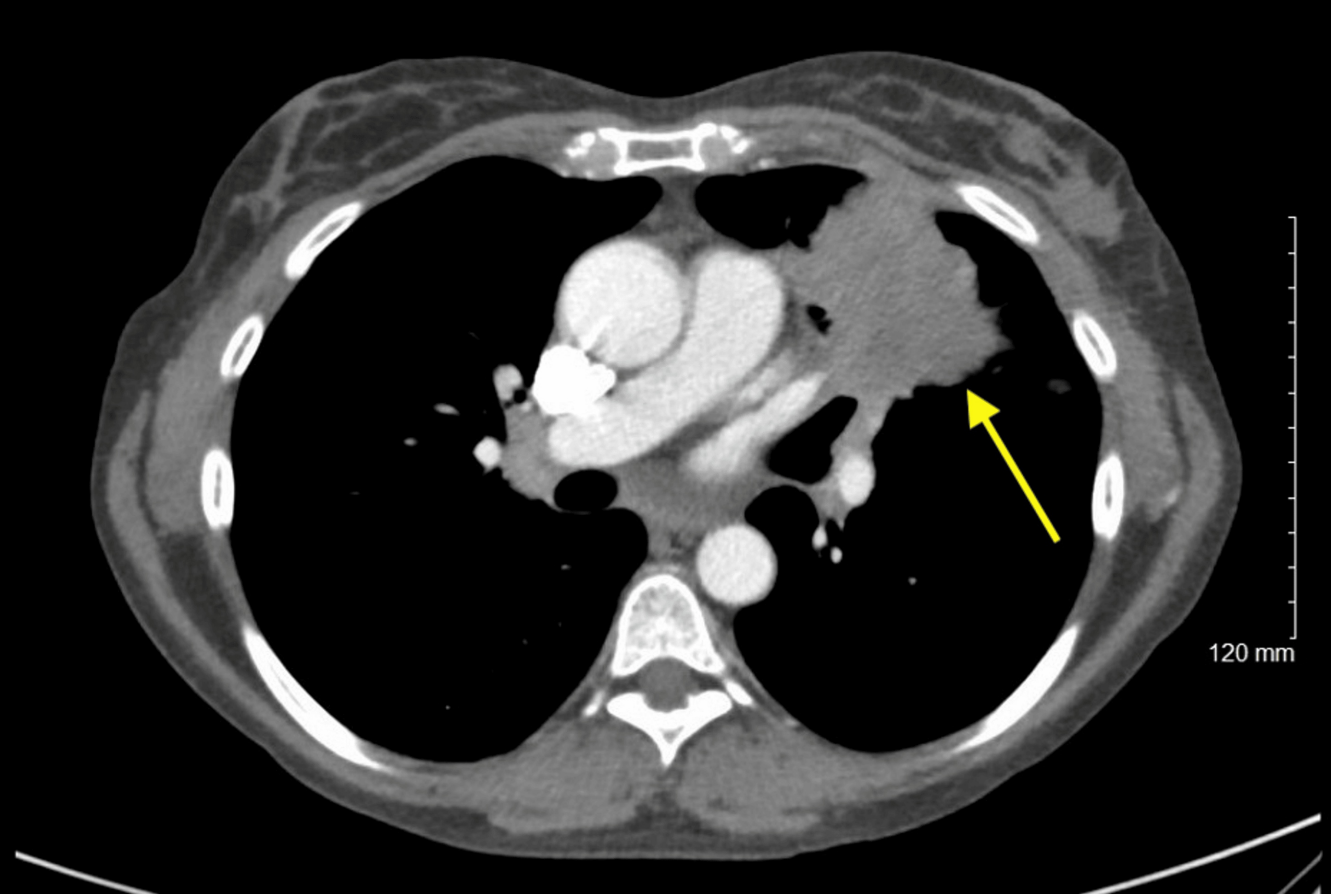
Axial CT of the chest with contrast showing the left upper lobe lung mass with mediastinal invasion

CT chest showed a 5.3 cm left upper lobe lung mass abutting the mediastinum and occluding the bronchial segments of the left upper lobe and lingula, along with left hilar, abdominal and retroperitoneal lymphadenopathy, and metastases to the bilateral adrenal glands and spleen (Figure 3).

**Figure 4:**
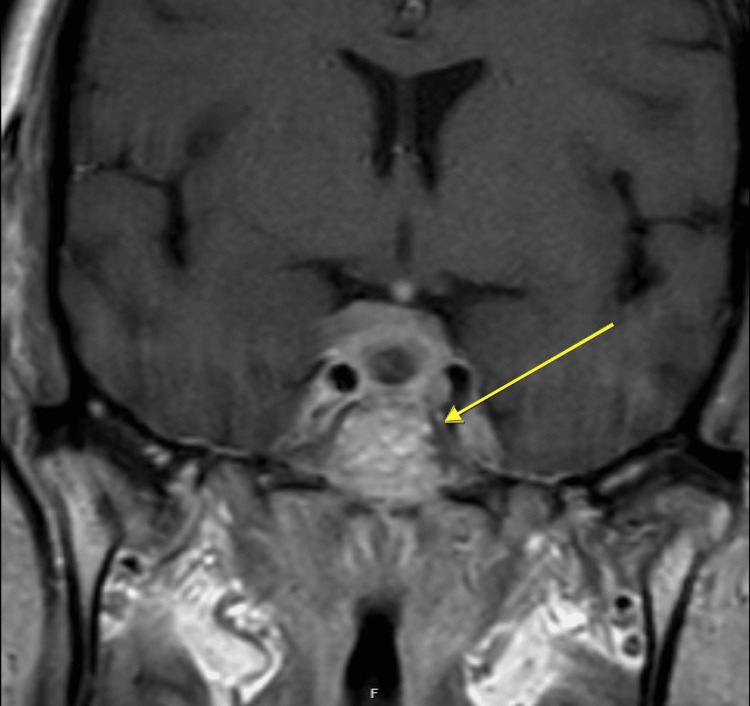
Coronal T1 MRI of the orbits with intravenous gadolinium contrast and fat suppression series demonstrating residual tumor above the sella turcica with invasion into the right cavernous sinus

The differential diagnosis for the sellar/suprasellar mass was concerning for pituitary adenoma vs. metastasis of primary lung cancer vs. meningioma, with vision loss attributed to compression and less likely ischemia. She underwent endoscopic endonasal transsphenoidal resection of the sellar tumor, during which a normal pituitary gland was noted beside the mass. Postoperative imaging revealed residual tumor above the sella turcica measuring 2.0 x 1.6 x 0.3 cm over the planum sphenoidale and redemonstration of cavernous sinus invasion on the right, with some tumor contacting the optic nerves (Figure 4).

**Figure 5:**
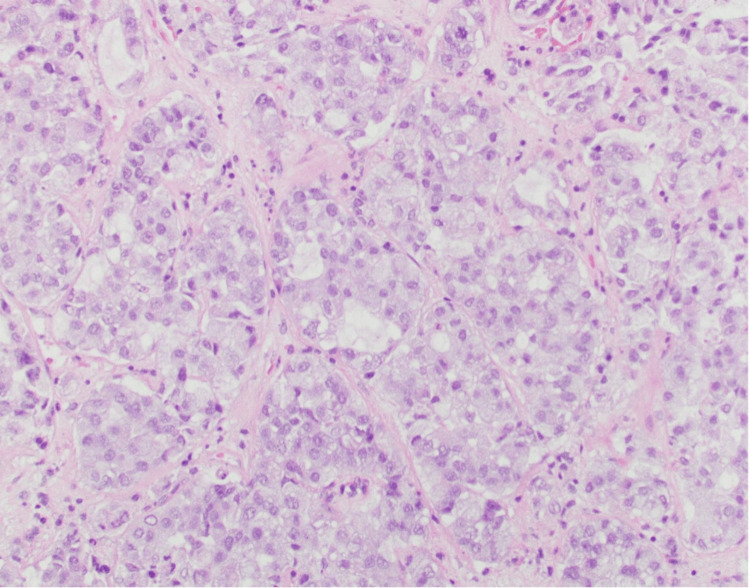
High-power image of metastatic adenocarcinoma in the pituitary gland biopsy demonstrating variably sized malignant glands and solid nests of malignant epithelial cells (hematoxylin and eosin stain, 20× magnification). No normal pituitary gland is present in this image

**Figure 6:**
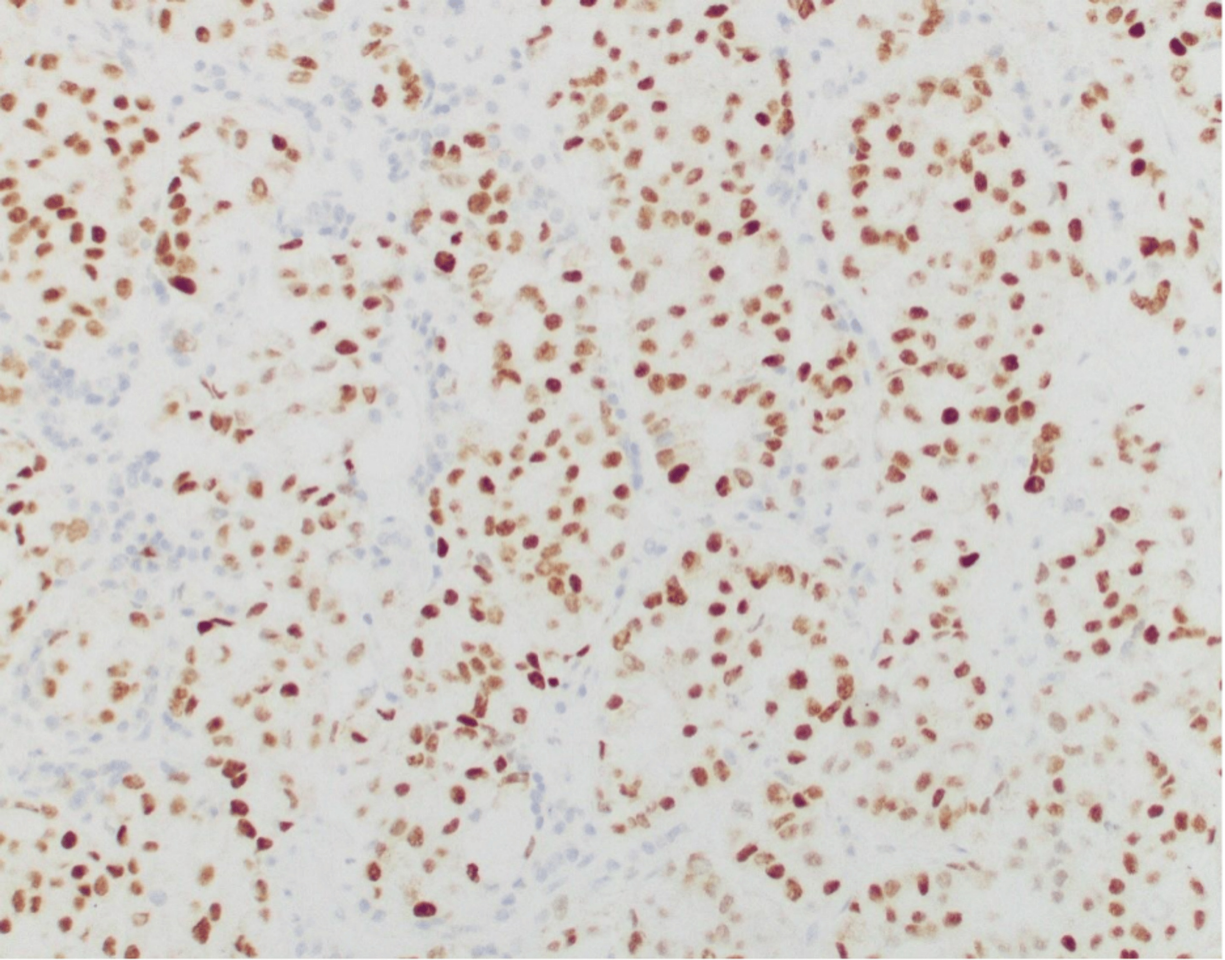
Image of TTF-1 (clone 8G7G3/1, Ventana, Roche Diagnostics, Oro Valley, Arizona) immunohistochemically stained section of the metastatic adenocarcinoma in the pituitary gland biopsy, demonstrating diffuse strong positive nuclear staining of tumor cells (20x magnification) TTF-1: thyroid transcription factor-1

Surgical pathology of the tissue biopsy from the pituitary mass showed thyroid transcription factor-1 positive metastatic adenocarcinoma consistent with lung primary (Figures 5, 6), with programmed death-ligand 1 (PDL1) score of 1%-10%, solid gene panel positive for pathogenic KRAS variant (p.G12C) and concomitant STK11 variant predicted to result in loss of function (STK11 p.Y166). The patient was diagnosed with stage IVB, T4 N1 M1c non-small cell lung cancer (NSCLC) with metastatic adenocarcinoma to the pituitary gland.

The patient was treated with dexamethasone for secondary adrenal insufficiency, levothyroxine for central hypothyroidism, and desmopressin for transient diabetes insipidus. She continued to require chronic steroids and thyroid hormone replacement. Postoperatively, she had no improvement in vision, and two weeks after the resection, she experienced new left-sided vision loss. Reimaging two weeks postoperatively showed a stable pituitary lesion and a new punctate left frontal metastasis. Radiation therapy began three weeks postoperatively with hypofractionated radiation to the pituitary metastasis, at a total dose of 2,500 cGy over five daily fractions given via a volumetric modulated arc therapy technique. Following the five-day course of radiation, she received systemic therapy with carboplatin, pemetrexed, and pembrolizumab every three weeks.

In the weeks after receiving radiation, she reported improvement in vision in her left eye. Reimaging four months after diagnosis showed a decrease in the sellar/suprasellar mass, stability of the left frontal lobe lesion, decrease in lung mass and hilar nodal tissue, normalization of the adrenal glands, decrease in the splenic lesion, and resolution of abdominal lymphadenopathy consistent with good response to therapy. However, she also developed hepatomegaly, transaminitis, and moderate to severe hepatic steatosis in the setting of binge drinking.

Due to a relapse of her chronic intermittent alcoholism, she required inpatient rehabilitation and missed two cycles of systemic treatment. Her liver function tests normalized after alcohol cessation and the resumption of chemotherapy.

This patient received surgical resection, sellar radiation, and systemic chemoimmunotherapy, along with desmopressin and hormonal replacement. Other treatment options could include optic chiasm decompression and intrathecal chemotherapy. This patient's postoperative left-sided vision loss mildly improved after radiation and systemic therapy. However, she was lost to ophthalmologic follow-up despite appointment reminders, and thus, formal visual field testing was not performed following the completion of her treatment.

## Discussion

We present a rare case of adenocarcinoma of the lung with symptomatic metastasis to the pituitary gland. This patient presented with vision loss, presumably secondary to malignant compression of the optic nerve. Her reported nausea and headaches may be attributed to intracranial hypertension secondary to the pituitary mass. Her chills and night sweats are likely due to systemic manifestations of malignancy.

Clinical features

She was found to have diabetes insipidus due to posterior lobe dysfunction. Her hyperprolactinemia, central hypothyroidism, and adrenal insufficiency are due to anterior lobe dysfunction. In an analysis of 201 patients with PMs, 50.6% of PMs involved the neurohypophysis alone, 15.4% infringed upon the adenohypophysis alone, and 33.8% destroyed both the anterior and posterior pituitary glands [1,4].

Diagnosis

PMs are increasing in frequency secondary to improved survival rates for cancer patients and more sensitive imaging. However, PMs are often too small for radiologic diagnosis. They are difficult to distinguish clinically and radiologically from primary sellar tumors, such as pituitary nonfunctioning adenomas or secretory adenomas (prolactin, adrenocorticotropic hormone, and growth hormone), as well as from granulomas, abscesses, cysts, aneurysms, trauma, or apoplexy. Symptomatic PMs are recently reported in 2.5%-18.2% of all cases; anterior pituitary deficiency may be masked in end-stage cancer patients with systemic complications of malignancy [5]. When symptomatic, patients most frequently present with diabetes insipidus, aligning with the propensity of metastasis to the posterior lobe via the hypophyseal arteries or direct contact with the dura. Diabetes insipidus occurs in less than 1% of pituitary adenoma cases and can therefore aid in differentiating PMs from pituitary adenomas [6]. The anterior lobe is supplied by the hypothalamohypophyseal portal system, a capillary network that supplies the hypothalamus, though it is also partially supplied by the superior hypophyseal artery. Other possible symptoms and associated conditions include secondary hypothyroidism, hypoadrenalism, hypogonadism, hyperfunctional syndromes (i.e., Cushing's syndrome, acromegaly, and syndrome of inappropriate antidiuretic hormone secretion), visual field impairment (bilateral hemianopsia), cranial nerve III palsy, cranial nerve IV palsy, cognitive deficit, psychiatric symptoms, anosmia (cranial nerve I), headaches, intracerebral hemorrhage, and hyperprolactinemia [7].

Molecular testing

One case study demonstrates complete resolution of progressive sellar disease from metastatic non-small cell lung cancer with osimertinib, a third-generation epidermal growth factor receptor (EGFR)-tyrosine kinase inhibitor, after discontinuation of carboplatin and pemetrexed due to an unfavorable side effect profile [8]. This case suggests that targeted therapy, specifically tyrosine kinase inhibitors in cases with mutant EGFR, can potentially spare patients the need for radiation therapy [8]. As is the case with the presented patient, tumor findings of mutant KRAS and the loss of STK11 confer a poor prognosis [9]. Furthermore, KRAS and STK11 are among the top mutated genes in NSCLC brain metastases [9]. However, when analyzing the top 10 most frequently mutated genes, about 37% were discordant between the genetic markers in the primary NSCLC tumor and the brain metastasis, thus indicating the need for molecular testing on both tissue types [9]. It has been reported that the brain metastases tend to have low PDL-1 expression, thus suggesting that immunotherapy with immune checkpoint inhibitors may have limited utility in these cases [9].

Prognosis and treatment

Treatment plans for PMs are tailored for each patient based on their primary cancer, presenting symptoms, patient condition, and tumor markers. Surgical resection is favored for patients with symptoms of pituitary compression (headache, visual field loss, visual acuity loss, and eye muscle paralysis), as was seen here in the presented patient [10]. Complete resection is difficult due to the aforementioned complex blood supply of the pituitary gland as well as robust tumor vascularity. Therefore, radiation, systemic chemotherapy, and other targeted therapies, including immunotherapy, are often included in the treatment plan. Multidisciplinary treatment can help improve the quality and length of life, though the prognosis of metastatic cancer with pituitary metastases remains poor [10]. A review of 41 cases of PMs reports prolonged survival in patients who undergo surgical resection, thus suggesting a benefit to surgery other than palliation [11]. The median survival is reported to be 14 months. Patients who underwent surgical resection had a median survival of 18 months compared to nine months for those who did not have surgery [11]. Due to the rarity of these cases and small sample sizes, more research is needed to better determine the most effective treatments to effectively reduce symptoms and improve prognosis.

This is a case of a woman with no prior oncologic history who presented with unilateral vision loss; she was found to have central hypothyroidism, secondary adrenal insufficiency, and transient diabetes insipidus; she was ultimately diagnosed with primary lung adenocarcinoma with metastasis to the pituitary gland, adrenal glands, abdominal and retroperitoneal lymph nodes, and spleen. She had a good response to therapy; however, her vision loss progressed postoperatively to involve both eyes, in part due to the time elapsed between the onset of visual loss and her presentation/treatment. The loss was partially reversed following radiation and systemic therapy. The timing and coordination of best management can be difficult, especially in rural healthcare settings and those with limited resources. The above-mentioned symptoms, even in isolation, should prompt imaging and further workup in a time-sensitive manner to prevent permanent deficits. Molecular testing of the primary tumor and metastasis can further help guide treatment. Though ultimately carrying a poor prognosis, early consideration of metastatic disease in the differential diagnosis of pituitary masses has the potential to improve patient outcomes and preserve organ function. Multidisciplinary collaboration and intervention are key to maximizing treatment efficacy and subsequently providing increased quality of life for this patient population.

## Conclusions

This is a case of a woman with no prior oncologic history who presented with unilateral vision loss, was found to have central hypothyroidism, secondary adrenal insufficiency, and transient diabetes insipidus, and was ultimately diagnosed with primary lung adenocarcinoma with metastasis to the pituitary gland, adrenal glands, abdominal and retroperitoneal lymph nodes, and spleen. She had a good response to therapy; however, her vision loss progressed postoperatively to involve both eyes, in part due to the time elapsed between the onset of visual loss and her presentation/treatment. The loss was partially reversed following radiation and systemic therapy. The above mentioned symptoms, even in isolation, should prompt imaging and further work up in a time sensitive manner to prevent permanent deficits. Though ultimately carrying a poor prognosis, early consideration of metastatic disease in the differential diagnosis of pituitary masses has the potential to improve patient outcomes and preserve organ function. In patients presenting with new visual loss along with symptoms of pituitary dysfunction, specifically diabetes insipidus, metastatic disease should be considered, even in the absence of known primary malignancy. In cases such as this, multidisciplinary collaboration, including endocrinology, neuro-ophthalmology, oncology, radiation oncology, surgical oncology, and neurosurgery, is key to maximizing treatment efficacy and subsequently providing increased quality of life.
